# Potential value of the homologous recombination deficiency signature we developed in the prognosis and drug sensitivity of gastric cancer

**DOI:** 10.3389/fgene.2022.1026871

**Published:** 2022-11-16

**Authors:** Xin Wu, Qiong Wang, Peifa Liu, Linde Sun, Yu Wang

**Affiliations:** ^1^ Department of General Surgical Medicine, The First Medical Center of PLA General Hospital, Beijing, China; ^2^ Pathology Department, The First Medical Center of PLA General Hospital, Beijing, China

**Keywords:** homologous recombination deficiency, gastric cancer, HRD scores, prognosis, Talazoparib

## Abstract

**Background:** Homologous recombination is an important DNA repair mechanism, which deficiency is a common feature of many cancers. Defining homologous recombination deficiency (HRD) status can provide information for treatment decisions of cancer patients. HRD score is a widely accepted method to evaluate HRD status. This study aimed to explored HRD in gastric cancer (GC) patients’ clinical outcomes with genes related to HRD score and HRD components score [HRD-loss of heterozygosity (LOH), large-scale state transitions (LST), and telomeric allelic imbalance (NtAI)].

**Methods:** Based on LOH, NtAI scores, LST, and integrated HRD scores-related genes, a risk model for stratifying 346 TCGA GC cases were developed by Cox regression analysis and LASSO Cox regression. The risk scores of 33 cancers in TCGA were calculated to analyze the relationship between risk scores of each cancer and HRD scores and 3 HRD component scores. Relationship between the risk model and patient survival, BRCA1, BRCA2 mutation, response to Cisplatin and Talazoparib treatment was analyzed by generating Kaplan-Meier curve, mutations waterfall map and conducting Pearson correlation analysis.

**Results:** An gene signature was constructed based on 11 HRD scores-related gene (BEX2, C1QL2, DKK1, DRC1, GLUD2, HCAR1, IGFBP1, NXPH1, PROC, SERPINA5, and SLCA1A2). Risk groups were stratified by risk score. Prognosis of the high-risk score group was worse than the low-risk ones. Risk score was associated with BRCA2 mutation, and patients grouped according to BRCA2 mutation status had distinguishable risk score, NtAI score, HRD-LOH, LST, and HRD scores. The low-score group showed higher sensitivity to Cisplatin and Talazoparib. The risk score of adrenocortical carcinoma (ACC), stomach adenocarcinoma (STAD), uterine corpus endometrial carcinoma (UCEC), kidney renal clear cell carcinoma (KIRC), sarcoma (SARC), prostate adenocarcinoma (PRAD), breast invasive carcinoma (BRCA) was significantly positively correlated with HRD score.

**Conclusion:** We developed an 11 HRD scores-related genes risk model and revealed the potential association between HRD status and GC prognosis, gene mutations, patients’ sensitivity to therapeutic drugs.

## Introduction

Gastric cancer (GC) is one of the most common and deadly cancers, with the fifth morbidity and the fourth mortality among all cancers. In 2020, more than 1 million new cases and nearly 769,000 deaths have been reported (Sung et al., 2021). Most GC are adenocarcinomas, originating from glands in the outermost layer of the stomach or mucosa (Cann and Ciombor, 2022). Helicobacter pylori infection, diets low in fruits and vegetables, high salt intake, age are Risk factors for the disease (Smyth et al., 2020). Although surgical resection, radiotherapy and chemotherapy, pressurized intraperitoneal aerosol chemotherapy (PIPAC), hyperthermic intraperitoneal chemotherapy (HIPEC) and other treatments have significantly improved the average 5-year survival rate of clinical patients, the average 5-year survival rate has reached 32%, which is still not ideal. The risk of residual lesions, micrometastases and disease recurrence is still very high. Once distant metastasis occurs, the survival rate of patients is very low, only 6% (Machlowska et al., 2020; Otaegi-Ugartemendia et al., 2022). GC is still an important focus of clinical, epidemiological and transformational research. Previous studies have identified several environmental and genetic risk factors as well as some susceptibility conditions (Karimi et al., 2014). However, there are still many gaps in our understanding of the drivers and pathological mechanisms of GC at the molecular level. The comprehensive characterization of GC molecular spectrum is very important for risk stratification, screening and personalized decision-making.

Gene mutations, chromosomal aberrations and epigenetic alterations are some of the genetic/epigenetic influences on GC pathogenesis (Chia and Tan, 2016). The emergence of genomic instability resulted from genetic mutations due to endogenously or exogenously caused DNA failures or damage during DNA damage repair may be a possible mechanism underlying cancer development. Normal cells protect cells from genomic instability by initiating a highly accurate DNA repair mechanism, thus preventing the accumulation of transformational mutations (Ali et al., 2021). In cancer, defects in DNA repair system could lead to the accumulation of genetic changes, causing genomic instability. Among them, homologous recombination deficiency (HRD), which results in impaired DNA double strand break repair, is considered to be the deadliest of all DNA repair defects (Wagener-Ryczek et al., 2021). HRD status plays an important role in driving the progression of cancer and leaves scars throughout the genome, grouped as telomeric allelic imbalance (TAI), large-scale state transitions (LST), HRD-loss of heterozygosity (LOH) (Ngoi and Tan, 2021). Defining HRD status can facilitate decision-making in treatment and help predict the clinical outcomes for some cancer patients (Stewart et al., 2022). At present, HRD score detection is a recognized method to evaluate the status of HRD (Shen et al., 2022), but there is no universally accepted gold standard. Myriad Genetic’s myChoice HRD has developed a HRD status assessment system based on the overall situation of the above three scars. FDA has approved it as a concomitant diagnosis of poly-adenosine diphosphate ribose polymerase (PARP) inhibitor nilapalil in the treatment of ovarian cancer (Jenner et al., 2016; Telli et al., 2016). Given the fact that the pattern of genomic instability caused by HRD may seem different in different tissue types, it is necessary to study the utility of this HRD score in other cancer types.

In this study, we defined the HRD state of GC based on the sum scores of LOH score, LST score, number of TAI (NtAI) score and three HRD components, identified important HRD-related genes by analyzing the prognosis and clinical correlation of HRD status-related genes, and developed a risk model to reveal the potential association between HRD status and GC prognosis, gene mutations and patients’ sensitivity to therapeutic drugs.

## Materials and methods

### Collection and preprocessing of clinical data

The latest stomach adenocarcinoma (STAD) data set was extracted from TCGA platform (https://tcga-data.nci.nih.gov/), and the cases with clinicopathological stage, survival time, status and mutation information were sorted out, and a total of 346 cases were obtained. And clinical sample data for an additional 32 cancer types were also downloaded from TCGA, and Table S1 listed the sample sizes included in each cancer. The RNA-seq of the case was standardized as Fragments Per Kilobase Million (FPKM) format. The RNA-seq and clinical data of GC cases from GSE66229 and GSE84437 datasets were from Gene Expression Omnibus (http://www.ncbi.nlm.nih.gov/geo/). After sorting out, GSE66229 and GSE84437 included 330 and 431 cases respectively.

### Acquisition and screening of HRD score-related genes

The combined HRD score and HRD component scores, including LOH, LST, and NtAI scores, were obtained by referring to the studies by Knijnenburg et al. (2018). The protein-coding genes (PCGs) were sorted out using gencode.v32.annotation.gff3.gz gene annotation information provided on GENCODE website (https://www.gencodegenes.org/#). The correlation between HRD score or HRD Component scores and PCG was calculated in the PCG expression profile of TCGA and the intersection was obtained to screen HRD-related genes.

### Enrichment analysis of HRD scores related genes by GO and KEGG

The R package WedGestaltR (v0.4.2) were performed to analyze the Gene Ontology and Kyoto Encyclopedia of Genes and Genomes (KEGG) pathways enriched by HRD score-related genes. The *p*-value adjusted by The Benjamini-Hochbergch procedure was regarded as the cutoff threshold. The results of the top 10 GO terms and top 5 KEGG pathways showed a bubble diagram.

### Screening of key HRD score related genes and construction of prognostic signature

To screen the genes significantly associated with overall survival (OS) from HRD-related genes, univariate Cox regression analysis was performed. Least absolute shrinkage and selection operator (LASSO) regression was performed to reduce dimensionality utilizing the “glmnet” package, and random forest regression was analyzed utilizing the “randomForestSRC” package. The HRD score-related genes screened by these analyses were used to construct the survival risk score model. The specificity and accuracy of survival prediction were evaluated by Kaplan-Meier (KM) curve and receiver operating characteristic (ROC) curve.

### Clinical correlation and mutation of prognostic signature

We investigated the relationship between risk score, HRD score, HRD component scores and clinical pathological features. First, the cases were stratified based on different clinical parameters (N stage, grade, T stage, AJCC stage, and M stage), and then the differences of risk score, HRD score and HRD component scores between groups under each clinical parameter were compared by Kruskal–Wallis test and Wilcox test. Additionally, a gene mutation waterfall map varying with risks score was generated according to the mutation data processed by mutect2 in TCGA.

### Drug correlation analysis of risk score

The sensitivity of Cisplatin and PARP inhibitor Talazoparib was predicted by “pRRophetic” R package, and the correlation between the sensitivity of the two drugs and risk score, HRD score, HRD component scores was determined by Pearson correlation analysis. In addition, Cisplatin and Talazoparib responses of cases based on risk score, HRD score and HRD component scores stratification were analyzed.

### Statistical analysis

All the statistical analyses of this study were performed by R software (version 4.0.2, https://www.rproject.org/). Evaluation of survival outcome by Log-rank test and Kaplan-Meier methods. The area under the curve (AUC) was calculated by receiver operating characteristic (ROC) curve. The relationship of continuous variables between the two groups was examined by non-parameter Wilcoxon rank-sum test, and the relationship among three or more groups was examined by Kruskal−Wallis test. Statistical significance was reached when the value of *p* was less than 0.05.

## Results

### Identification and functional analysis of HRD and HRD components related genes in GC

The genes whose correlation with HRD score and HRD component scores were more than 0.2 and *p* < 0.05 were identified by correlation analysis. 2231 HRD score-related genes, 1651 HRD-LOH score-related genes, 1660 LST score-related genes and 2,377 NtAI score-related genes were screened in TCGA. There were 1,264 common genes among all four types of HRD related genes (Supplementary Figure S1A). GO analysis showed that the 1,264 genes were associated with homologous recombination, reciprocal meiotic recombination, chromatin assembly and other GO biological processes (BP), but there was no statistically significant correlation (Supplementary Figure S1B). KEGG enrichment analysis showed that 1,264 HRD related genes were enriched in glycosylphosphatidylinositol (GPI)−anchor biosynthesis, complement and coagulation cascades, systemic lupus erythematosus, alcoholism, viral carcinogenesis, however, the enrichment of these HRD related genes in these KEGG pathways did not show statistical significance (Supplementary Figure S1C).

### Prognostic value of HRD score and HRD component scores in GC

Four indicators, overall survival (GC), disease-specific survival (DSS), disease-free interval (DFI), and progression-free interval (PFI), were employed to investigate the relationship between HRD score/HRD component scores and GC prognosis. Kaplan-Meier curve showed that patients with different scores could be distinguished according to HRD score and HRD component scores, but there was no significant difference in overall survival between high score group and low score group (Supplementary Figure S2). Kaplan-Meier analysis of the association between HRD score and HRD component scores and DSS showed that HRD score, HRD-LOH score, LST score and NtAI score were significantly correlated with bad DSS and PFI, respectively (Supplementary Figure S3, S5). Moreover, individuals with high LST had significantly shorter DFI than individuals with low LST (Supplementary Figure S4).

### Development of prognostic signature composed of key HRD score-related genes

**FIGURE 1:**
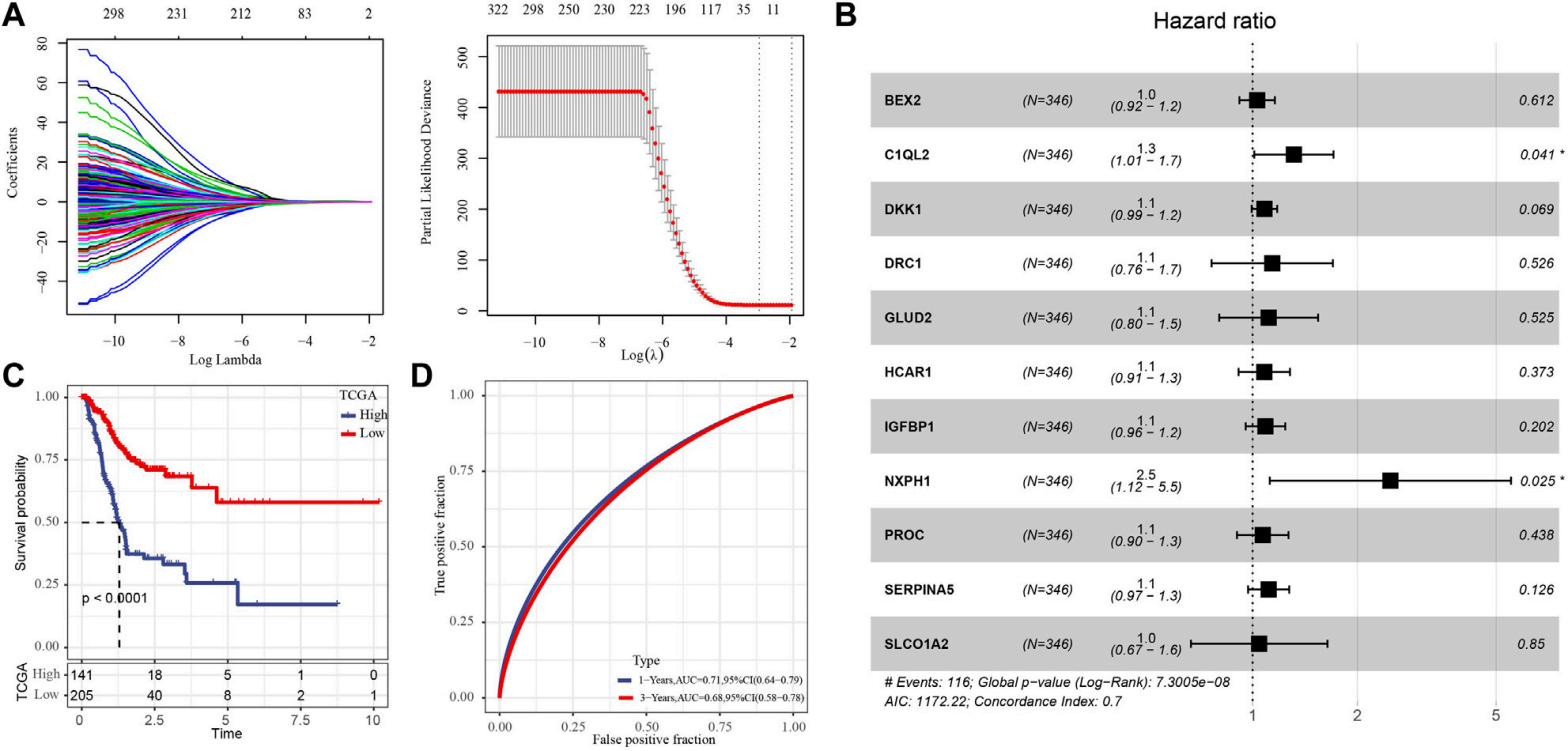
Development of prognostic signature composed of key HRD score-related genes. **(A)** The key genes were screened from 176 HRD score related genes by Lasso regression analysis. **(B)** Forest map of multivariate Cox regression analysis of 11 genes. **(C)** Kaplan-Meier survival analysis of for the high- and low-risk group in TCGA with 346 GC cases. **(D)** The ROC curve of the risk score of GC in TCGA.

Univariate Cox regression analysis showed that 176 of 1264 HRD score related genes were significantly associated with the survival of GC. Eleven genes were identified by Lasso regression analysis (Figure 1A). The regression coefficients of 11 genes were determined by multivariate Cox regression analysis (Figure 1B). The risk score of each GC case in TCGA was calculated by summing the product of regression coefficient and gene expression, the formula was:

$$\begin{array}{l} Risk\;score=0.0303*BEX2+0.2731*C1QL2+0.0784*DKK1\\ +0.1297*DRC1+0.1060*GLUD2\\ +0.0773*HCAR1+0.0851*IGFBP1\\ +0.9108*NXPH1+0.0674*PROC\\ +0.1061*SERPINA5+0.0436*SLCO1A2 \end{array}$$ (1)

We standardized the risk score by Z-score, and classified the samples into high-risk group and low-risk group with 0 as the boundary. And Survival analysis showed that the high-risk group had a worse prognosis than the low-risk individuals (Figure 1C). The ROC curve showed that the risk formula composed of 11 genes had a certain accuracy in predicting the survival of GC, with AUC of 0.71 and 0.68 in 1 year and 3 years, respectively (Figure 1D).

### Correlation between risk score and HRD score, HRD component scores and clinical features

**FIGURE 2:**
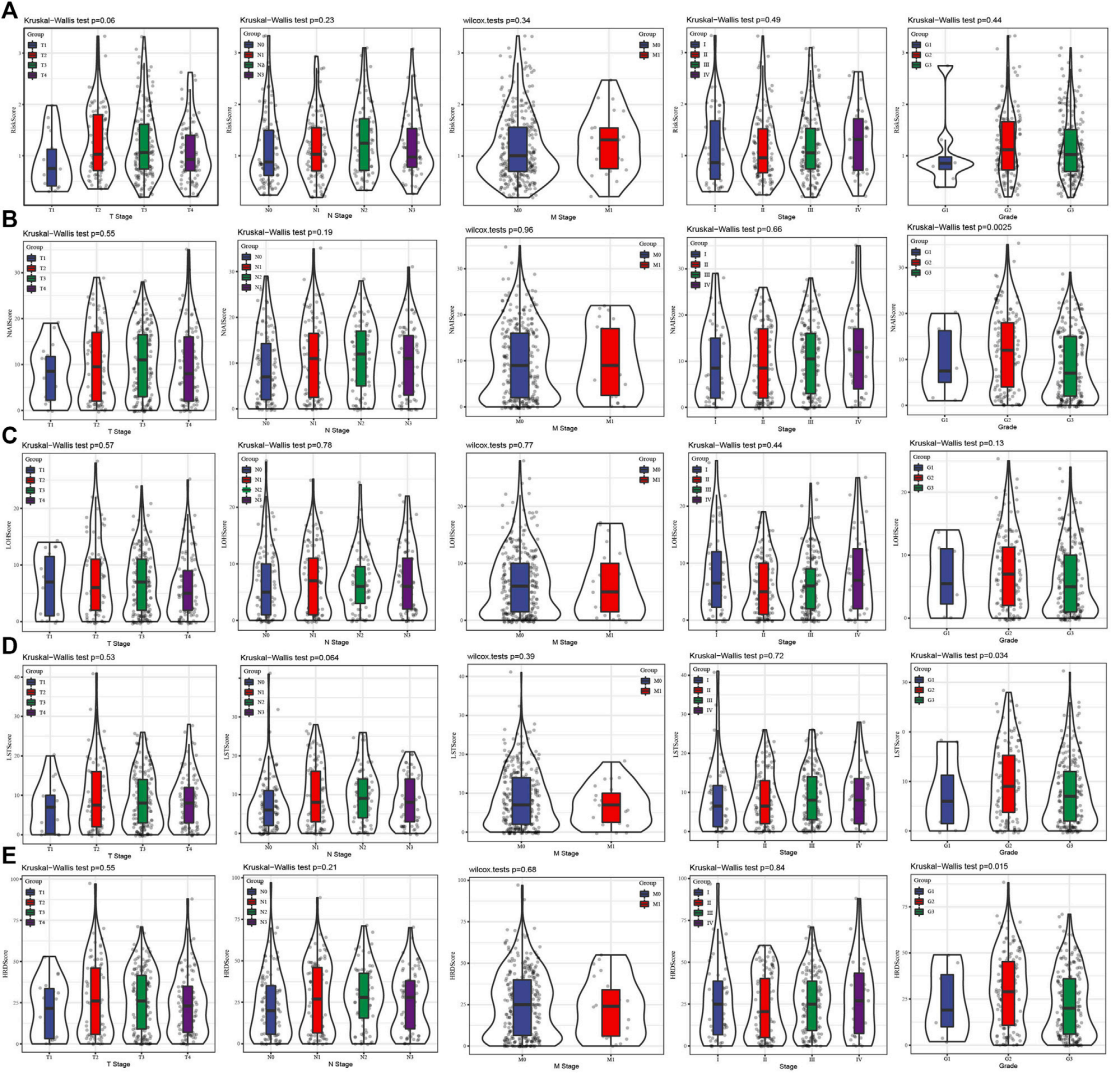
Association between risk score and different clinical stages and grade. **(A)** The risk score of the cases stratified by T stage, N stage, M stage, AJCC stage and grade. **(B)** The NtAI score of individuals stratified by T stage, N stage, M stage, AJCC stage and grade, respectively. **(C)** The correlation between LOH score and T stage, N stage, M stage, AJCC stage and grade. **(D)** LST score distribution in different T stage, N stage, M stage, AJCC stage and grade groups. **(E)** The association of HRD score with different T stage, N stage, M stage, AJCC stage and grade.

Through the Pearson correlation analysis to explore the relationship between risk score and HRD score, HRD component scores. The results showed that risk score was positively correlated with HRD score and three kinds of HRD component scores (Supplementary Figure S6). Then the relationship between risk score/HRD score/HRD component scores and several clinical stages and grade was analyzed. There was no significant difference in risk score between each clinical stage and grade group (Figure 2A). There was no significant correlation between NtAI score and T stage, N stage, M stage, and AJCC stage, but there were significant differences among the three grades (Figure 2B). The same situation was also found in the association analysis between LST score/HRD score and T, N, M stage, AJCC stage, and grade (Figures 2D,E). For LOH score, no correlation was found between it and the clinical parameters tested (Figure 2C). These results showed that NtAI score, LST score, and HRD score were significantly correlated with tumor grade.

### Gene mutation in prognostic signature

**FIGURE 3:**
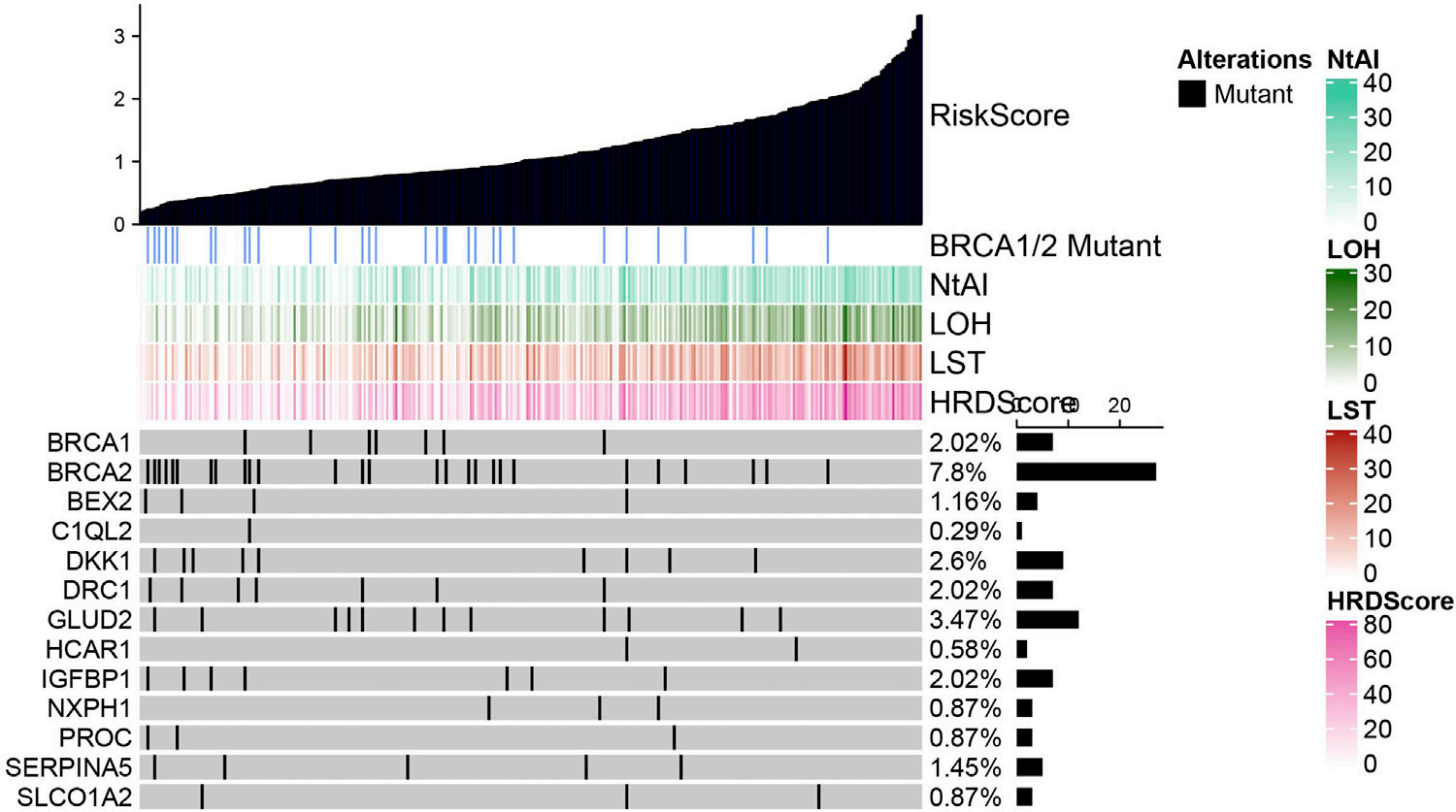
Mutation trend of BRCA1, BRCA2 and 11 HRD related genes under different risk score.

By plotting mutation waterfall maps of BRCA1, BRCA2, and 11 HRD-related genes in signature under different risk scores, the relationship between risk score and mutations of these genes was intuitively observed. Among all the genes displayed, the frequency of BRCA2 mutation was the highest, and mainly occurred in cases with lower risk score. With the increased of risk score, the frequency of mutation decreased gradually. In addition, HRD score and three HRD component scores also showed an increasing trend with the increase of risk score (Figure 3). The patients were divided into wild combination mutation group according to whether BRCA1 or BRCA2 was mutated. After analysis, it was found that risk score, NtAI score, HRD-LOH score, LST score, and HRD score did not show significant differences between BRCA1 wild type and mutant samples (Supplementary Figure S7A). Significant differences in risk score, NtAI score, HRD-LOH score, LST score, and HRD score were observed between BRCA2 wild type and mutant cases, specifically described as risk score, NtAI score, HRD-LOH, LST, and HRD score in BRCA2 mutant cases were significantly higher than those in BRCA2 wild type cases (Supplementary Figure S7B).

### Association between risk score/HRD score and drug sensitivity of GC therapy

**FIGURE 4:**
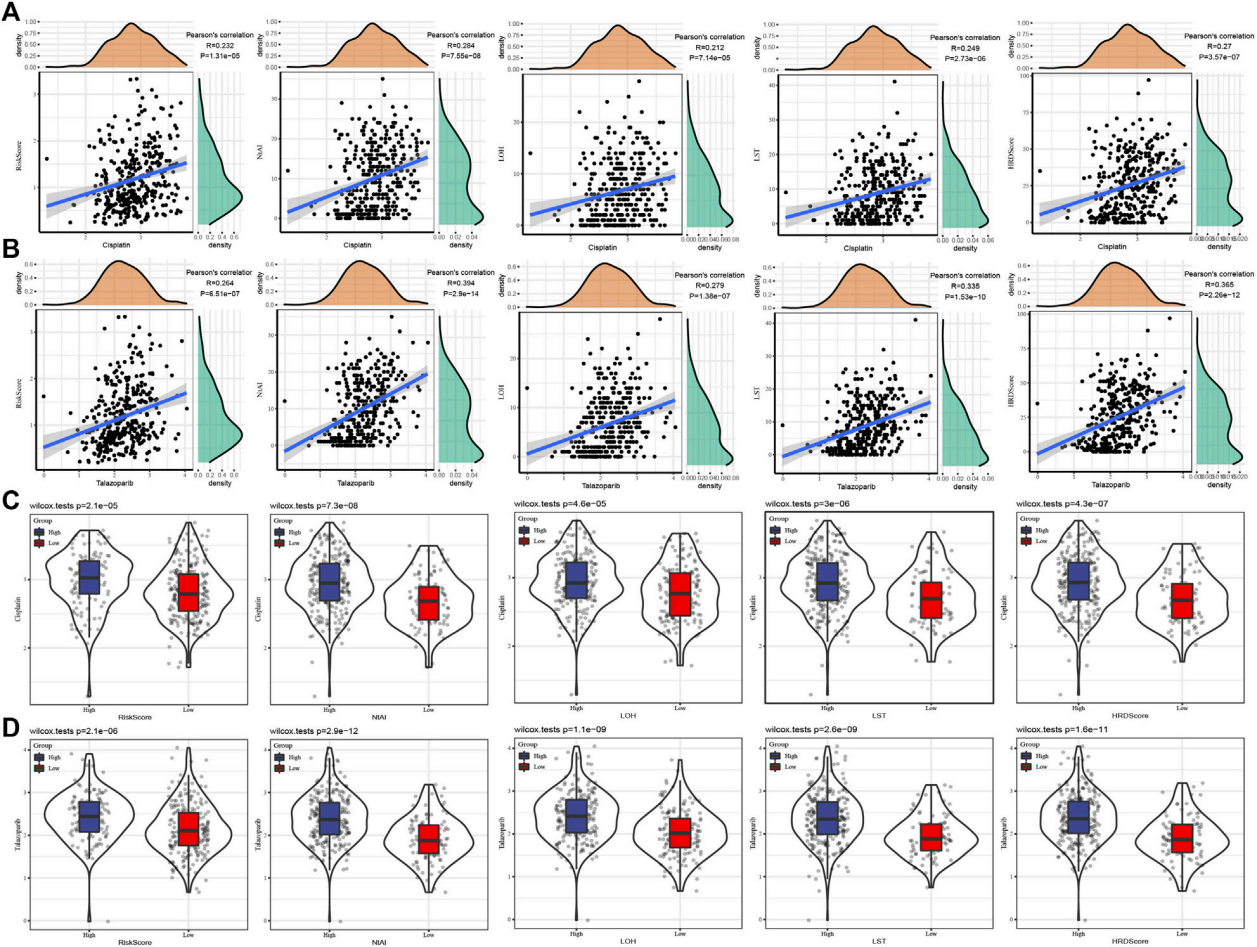
Association between risk score/HRD score and drug sensitivity of GC therapy. **(A)** The correlation between Cisplatin and risk score, HRD score, and HRD component scores was analyzed. **(B)** Pearson correlation analysis between Talazoparib and risk score, NtAI score, HRD-LOH score, LST score, and HRD score respectively. **(C)** Sensitivity to Cisplatin in GC cases grouped according to risk score, NtAI score, HRD-LOH score, LST score, and HRD score. **(D)** The IC50 values of Talazoparib in GC disease cases were divided into risk score, NtAI score, HRD-LOH score, LST score, and HRD score groups respectively.

To understand the relationship between risk score or HRD score and chemotherapy and targeted therapy, chemotherapeutic drug Cisplatin and targeted drug Talazoparib were selected to analyze their correlation with risk score/HRD score/HRD component scores. The results showed that Cisplatin and Talazoparib were positively correlated with risk score, NtAI score, HRD-LOH score, LST score, and HRD score, respectively (Figures 4A,B). No matter the cases were divided into risk groups according to risk score or cases grouped according to NtAI score, HRD-LOH score, LST score, and HRD score respectively, Cisplatin and Talazoparib always showed higher sensitivity than high score group in low score group (Figures 4C,D).

### The performance of prognostic signature was evaluated in the validation set

**FIGURE 5:**
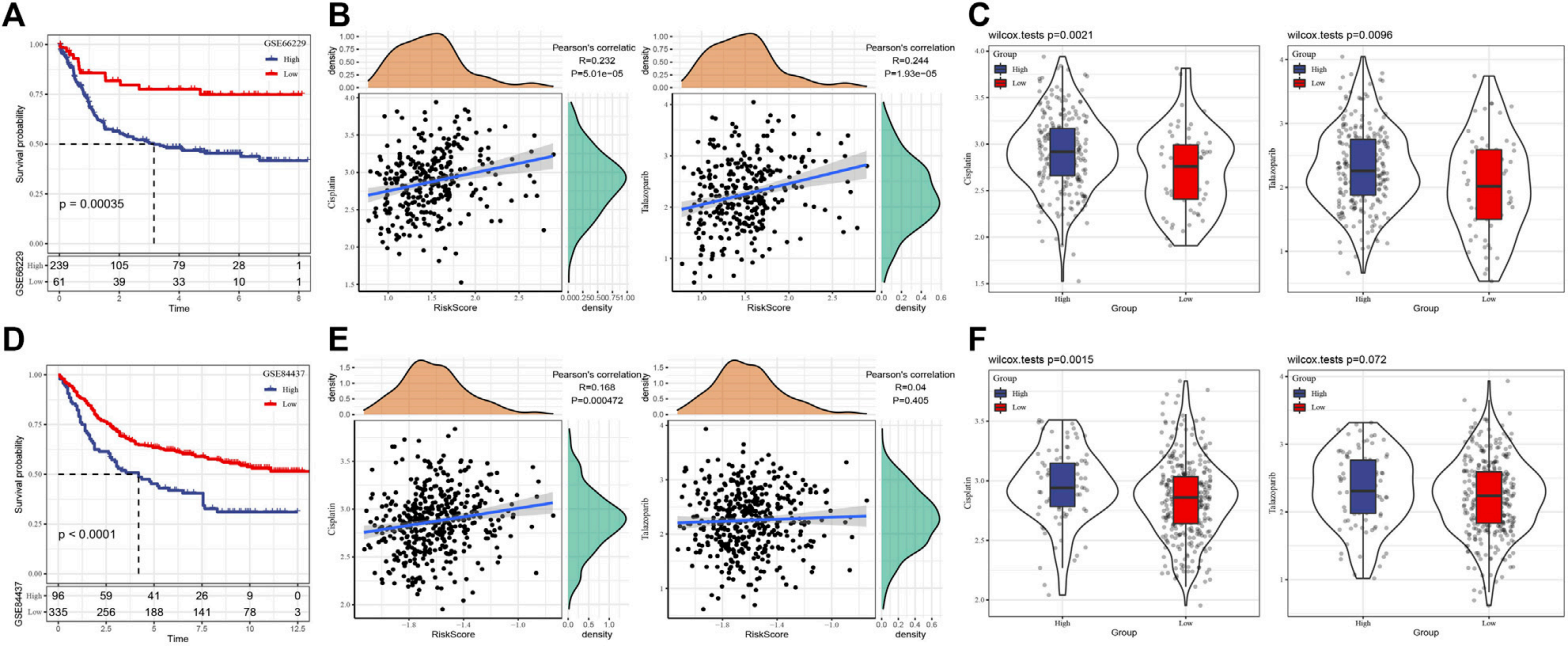
The performance of prognostic signature was evaluated in the validation set. **(A)** The survival curve of high-risk and low-risk groups in GSE66229 dataset. **(B)** Correlation between risk score and Cisplatin and Talazoparib in GSE66229 dataset. **(C)** Sensitivity of Cisplatin and Talazoparib in two risk groups of GSE66229 dataset. **(D)** Survival results of high-risk and low-risk groups in GSE84437 dataset. **(E)** Pearson correlation between risk score and Cisplatin and Talazoparib in GSE84437 datasets. **(F)** Response analysis of high-risk and low-risk groups to Cisplatin and Talazoparib in GSE84437 dataset.

For the signature constructed in TCGA based on 11 HRD-related genes, we evaluated its performance in two verification sets, GSE66229 and GSE84437. First of all, in two independent verification sets, the risk score of each case were generated and standardized by Z-score. Case with a risk score >0 was considered as a high-risk case, and with a risk score < 0 was defined as a high-risk case. The survival analysis showed that the survival time of high-risk cases in two independent verification sets was significantly shorter than that of low-risk patients (Figures 5A,D). The results of Pearson correlation analysis also showed that there was a significant positive correlation between risk score and Cisplatin and Talazoparib in each verification set (Figures 5B,E). The analysis of Cisplatin and Talazoparib sensitivity of patients in different risk groups in GSE66229 showed that the low-risk group was more sensitive to these two drugs (Figure 5C). In another verification set, both drugs had lower IC50 in the low-risk group, but there was no significant difference in the sensitivity of Talazoparib between the high-risk and low-risk groups (Figure 5F).

### Pan-cancer analysis of risk score

**FIGURE 6:**
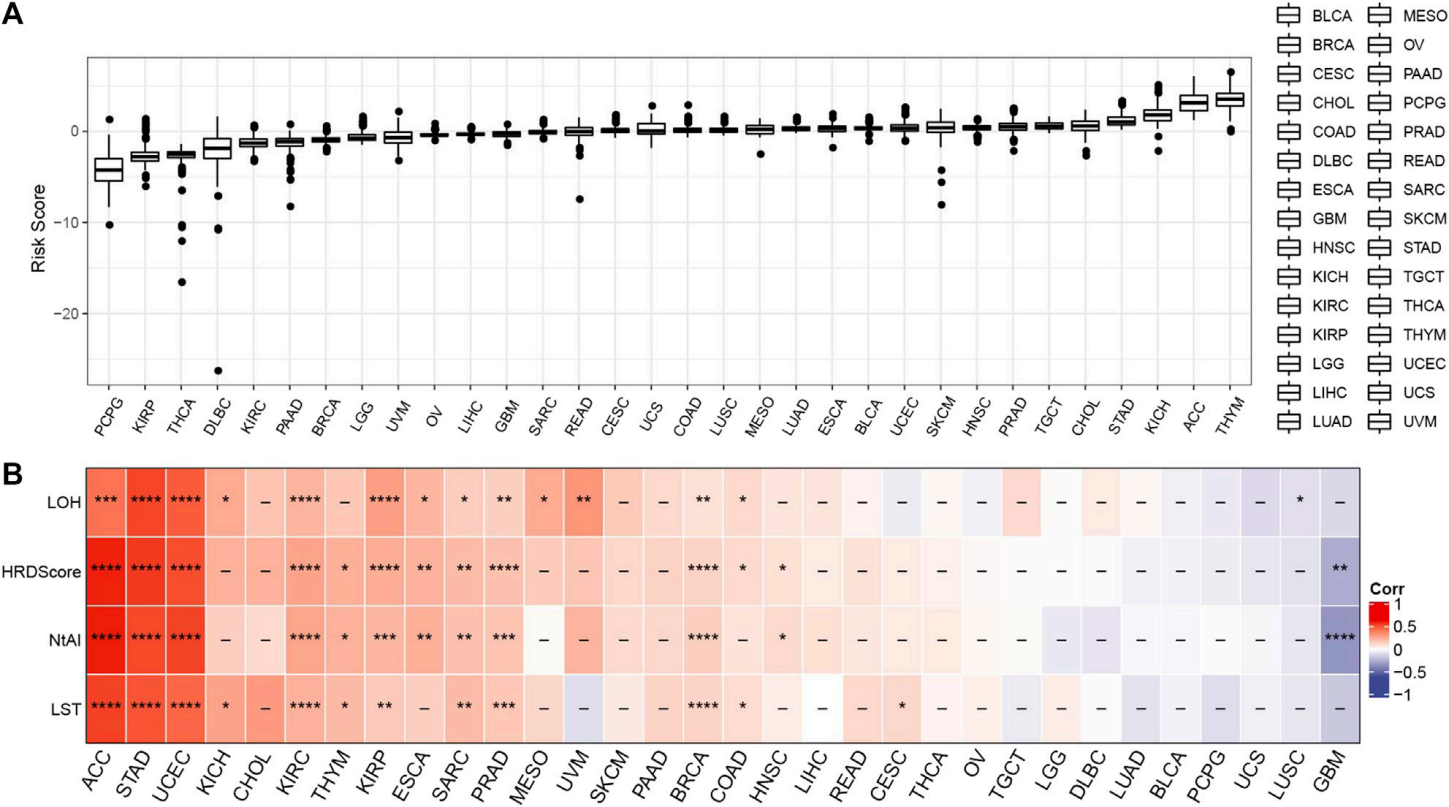
Pan-cancer analysis of risk score. **(A)** Risk score was distributed in each type of cancer tissue. **(B)** The correlation between risk score and HRD score and three kinds of HRD component scores in each kind of cancer. *p < 0.05, **p < 0.01, ***p < 0.001, ****p < 0.0001.

To explore the performance of risk score in different tumors, the risk score of solid tumors in TCGA was calculated according to the risk model. Figure 6A showed the distribution of risk score in each type of cancer tissue. In addition, the correlation between risk score and HRD score and three kinds of HRD component scores in each kind of cancer was studied. According to the results of Pearson correlation analysis, the risk score of ACC, STAD, UCEC, KIRC, SARC, PRAD, and BRC were significantly positively correlated with HRD score and three kinds of HRD component scores, indicating that the risk model may have a potential effect on the HRD of these cancers (Figure 6B).

## Discussion

HRD has been regarded as a marker of many cancers (Hoppe et al., 2018). Improved responses to platinum-based chemotherapy and PARP inhibitors have been observed in patients with HRD (da Cunha Colombo Bonadio et al., 2018). The analysis, verification and clinical identification of stratified biomarkers are essential for the use of these drugs to provide accurate patient care (Mateo et al., 2019). FDA-approved companion HRD assays could be applied for PARP inhibitor use, however, current HRD assays could not consistently filter a patient subgroup who could not gain benefit from PARP inhibitors, which might as well lead to potential resistance to PARP inhibitor therapy (Chiang et al., 2021). Here, we described a classifier based on HRDscore-related genes, which can classify GC patients by risk stratification, detect GC prognosis, HRD-related gene mutations, and help identify GC patients who benefit from PARP inhibitor Talazoparib therapy. Through the pan-cancer analysis of this tool, we reveal its potential for HRD prediction of different cancers.

The combination of multiple biomarkers challenges some traditional concepts of biomarker verification in the development of anticancer drugs (Mateo et al., 2019). The risk model developed in this study was based on the combination of 11 HRD score and HRD components score related genes. Brain ‐expressed X‐linked gene 2(BEX2) has been found to repair dormant cancer stem cells in liver cancer (Tamai et al., 2020; Fukushi et al., 2021), and has shown cancer-promoting activity in several cancers (Naderi et al., 2007; Tan et al., 2020). As an inhibitor of Wnt signal pathway, dickkopf-1 (DKK1) has been proved to be an independent risk factor in ESCA, LUAD, MESO and STAD (Gao et al., 2021). The expression of glutamate dehydrogenase 2 (GLUD2) is associated with the histopathological classification, prognosis and survival of patients with glioblastoma. Up-regulation of its expression resulted in the inhibition of glioblastoma cell growth (Franceschi et al., 2018). Hydroxycarboxylic acid receptor 1 (HCAR1) was previously reported to be involved in the enhancement of DNA repair in cervical cancer cells related to lactic acid. The expression of lactic acid receptor/HCAR1 helps to regulate the mechanism of DNA repair in cervical cancer cells (Wagner et al., 2017). Study reported that Insulin-like growth factor binding protein-1 (IGFBP-1) encodes a secretory protein associated with the risk of a variety of tumors, including in breast cancer, liver cancer, gastrointestinal cancer and endometrial cancer (Lin et al., 2021). An immunohistochemical study based on pancreatic ductal adenocarcinoma showed a negative correlation between neurexophillin-1 (NXPH1) and T stage of the tumor. The detection of NXPH1 may be help delineate appropriate surgical margins, and identify lymph node metastasis in imaging studies (Jin and Tsai, 2016). The expression level of SERPINA5 was negatively correlated with the malignant progression of HCC, and this gene can regulate metastasis potential of hepatoma cells *in vitro* and *in vivo* (Jing et al., 2014). In triple negative breast cancer, SLCO1A2 encodes organic anion-transporting polypeptide 1A2 (OA TP1A2), and the expression of OATP1A2 and organic cation transporter 6 was predicted to be an indicator of response to neoadjuvant chemotherapy (Hashimoto et al., 2014). Although the role of these genes in different types of cancer has been studied, the association between their risk models and HRD has not been reported.

We confirmed the positive correlation between risk model and HRD score and three kinds of HRD component scores, and analyzed the relationship between risk score, HRD score, three kinds of HRD components and clinical stage and stage respectively. In DNA repair-defective tumors, genetic alterations have been shown to be able to reflect the scars resulted from using backup DNA repair mechanisms, but this needs to maintain cellular viability (Setton et al., 2021). Mutations of BRCA2 or BRCA1 genes are considered to be the most common signs of HRD (Gulhan et al., 2019). In addition to the harmful mutations of HRD -related genes such as BRCA1/2, we also explored the mutations of HRD score-related genes in risk models. In GC, the frequency of BRCA2 mutation was very high, and significant differences in risk score, NtAI score, HRD-LOH score, LST score, and HRD score were observed between wild type and mutant cases of BRCA2. More importantly, risk score could help identify patients who benefit from Cisplatin and Talazoparib treatment. And risk score also had a significant positive correlation with ACC, STAD, UCEC, KIRC, SARC, PRAD, and BRC, indicating its potential effect on the HRD of these cancers.

In summary, we developed a risk model based on HRD score-related genes, which can predict the prognosis of GC patients through risk stratification and help identify GC patients who benefit from PARP inhibitor Talazoparib therapy. Through the pan-cancer analysis of this tool, we provided new insights into the potential HRD status in different types of cancer.

## Data Availability

The original contributions presented in the study are included in the article/Supplementary Material, further inquiries can be directed to the corresponding author.

## Supplementary material

SUPPLEMENTARY FIGURE S1 Identification and functional analysis of HRD and HRD components related genes in GC : Overlap analysis of HRD score and HRD component scores related genes. **(B)**: GO analysis of 1264 HRD-related genes showed that top 10 GO biological processes (BP) and GO cellular components (CC) and GO molecular function enriched by HRD-related genes were shown in the bubble diagram. **(C)**: Top 5 KEGG pathways enriched by 1264 HRD related genes.

SUPPLEMENTARY FIGURE S2 The correlation between HRD score, HRD-LOH score, LST score, NtAI score and overall survival.

SUPPLEMENTARY FIGURE S3 Disease-specific survival (DSS) analysis of cases grouped according to HRD score, HRD-LOH score, LST score, and NtAI score.

SUPPLEMENTARY FIGURE S4 The relationship between HRD score and HRD component scoresand disease-free interval (DFI).

SUPPLEMENTARY FIGURE S5 Individual progression-free interval (PFI) survival curves were stratified according to HRD score and different HRD component scores.

SUPPLEMENTARY FIGURE S6 Pearson correlation analysis between risk score and HRD score, LOH score, NtAI score and LST score.

SUPPLEMENTARY FIGURE S7 Risk score, NtAI, HRD-LOH, LST, and HRD score between BRCA1 and BRCA2 **(B)** wild type and mutant cases.

## References

[B1] Ali R. M. M. McIntosh S. A. Savage K. I. (2021). Homologous recombination deficiency in breast cancer: implications for risk, cancer development, and therapy. Genes Chromosom. Cancer 60 (5), 358–372. 10.1002/gcc.22921 33247475

[B2] Cann C. Ciombor K. K. (2022). Systemic therapy for gastric cancer: perioperative strategies and beyond. J. Surg. Oncol. 125, 1151–1160. 10.1002/jso.26834 35230696

[B3] Chia N. Y. Tan P. (2016). Molecular classification of gastric cancer. Ann. Oncol. 27 (5), 763–769. 10.1093/annonc/mdw040 26861606

[B4] Chiang Y. C. Lin P. H. Cheng W. F. (2021). Homologous recombination deficiency assays in epithelial ovarian cancer: Current status and future direction. Front. Oncol. 11, 675972. 10.3389/fonc.2021.675972 34722237 PMC8551835

[B5] da Cunha Colombo Bonadio R. R. Fogace R. N. Miranda V. C. Diz M. (2018). Homologous recombination deficiency in ovarian cancer: a review of its epidemiology and management. Clinics 73, e450s. 10.6061/clinics/2018/e450s 30133561 PMC6096977

[B6] Franceschi S. Corsinovi D. Lessi F. Tantillo E. Aretini P. Menicagli M. (2018). Mitochondrial enzyme GLUD2 plays a critical role in glioblastoma progression. EBioMedicine 37, 56–67. 10.1016/j.ebiom.2018.10.008 30314897 PMC6284416

[B7] Fukushi D. Shibuya-Takahashi R. Mochizuki M. Fujimori H. Kogure T. Sugai T. (2021). BEX2 is required for maintaining dormant cancer stem cell in hepatocellular carcinoma. Cancer Sci. 112 (11), 4580–4592. 10.1111/cas.15115 34424582 PMC8586677

[B8] Gao S. Jin Y. Zhang H. (2021). Pan-cancer analyses reveal oncogenic and immunological role of dickkopf-1 (DKK1). Front. Genet. 12, 757897. 10.3389/fgene.2021.757897 34899842 PMC8654726

[B9] Gulhan D. C. Lee J. J. Melloni G. E. M. Cortes-Ciriano I. Park P. J. (2019). Detecting the mutational signature of homologous recombination deficiency in clinical samples. Nat. Genet. 51 (5), 912–919. 10.1038/s41588-019-0390-2 30988514

[B10] Hashimoto Y. Tatsumi S. Takeda R. Naka A. Ogane N. Kameda Y. (2014). Expression of organic anion-transporting polypeptide 1A2 and organic cation transporter 6 as a predictor of pathologic response to neoadjuvant chemotherapy in triple negative breast cancer. Breast Cancer Res. Treat. 145 (1), 101–111. 10.1007/s10549-014-2913-y 24671357

[B11] Hoppe M. M. Sundar R. Tan D. S. P. Jeyasekharan A. D. (2018). Biomarkers for homologous recombination deficiency in cancer. J. Natl. Cancer Inst. 110 (7), 704–713. 10.1093/jnci/djy085 29788099

[B12] Jenner Z. B. Sood A. K. Coleman R. L. (2016). Evaluation of rucaparib and companion diagnostics in the PARP inhibitor landscape for recurrent ovarian cancer therapy. Future Oncol. 12 (12), 1439–1456. 10.2217/fon-2016-0002 27087632 PMC4976841

[B13] Jin J. S. Tsai W. C. (2016). The detection of tumor location and lymph node metastasis by aberrant NXPH1 and NXPH2 expressions in pancreatic ductal adenocarcinomas. Chin. J. Physiol. 59 (6), 348–354. 10.4077/CJP.2016.BAF430 27817196

[B14] Jing Y. Jia D. Wong C. M. Oi-Lin Ng I. Zhang Z. Liu L. (2014). SERPINA5 inhibits tumor cell migration by modulating the fibronectin-integrin β1 signaling pathway in hepatocellular carcinoma. Mol. Oncol. 8 (2), 366–377. 10.1016/j.molonc.2013.12.003 24388360 PMC5528558

[B15] Karimi P. Islami F. Anandasabapathy S. Freedman N. D. Kamangar F. (2014). Gastric cancer: descriptive epidemiology, risk factors, screening, and prevention. Cancer Epidemiol. Biomarkers Prev. 23 (5), 700–713. 10.1158/1055-9965.EPI-13-1057 24618998 PMC4019373

[B16] Knijnenburg T. A. Wang L. Zimmermann M. T. Chambwe N. Gao G. F. Cherniack A. D. (2018). Genomic and molecular landscape of DNA damage repair deficiency across the cancer genome atlas. Cell Rep. 23 (1), 239–254. 10.1016/j.celrep.2018.03.076 29617664 PMC5961503

[B17] Lin Y. W. Weng X. F. Huang B. L. Guo H. P. Xu Y. W. Peng Y. H. (2021). IGFBP-1 in cancer: expression, molecular mechanisms, and potential clinical implications. Am. J. Transl. Res. 13 (3), 813–832. 33841624 PMC8014352

[B18] Machlowska J. Baj J. Sitarz M. Maciejewski R. Sitarz R. (2020). Gastric cancer: epidemiology, risk factors, classification, genomic characteristics and treatment strategies. Int. J. Mol. Sci. 21 (11), E4012. 10.3390/ijms21114012 PMC7312039 32512697

[B19] Mateo J. Lord C. J. Serra V. Tutt A. Balmana J. Castroviejo-Bermejo M. (2019). A decade of clinical development of PARP inhibitors in perspective. Ann. Oncol. 30 (9), 1437–1447. 10.1093/annonc/mdz192 31218365 PMC6771225

[B20] Naderi A. Teschendorff A. E. Beigel J. Cariati M. Ellis I. O. Brenton J. D. (2007). BEX2 is overexpressed in a subset of primary breast cancers and mediates nerve growth factor/nuclear factor-kappaB inhibition of apoptosis in breast cancer cell lines. Cancer Res. 67 (14), 6725–6736. 10.1158/0008-5472.CAN-06-4394 17638883

[B21] Ngoi N. Y. L. Tan D. S. P. (2021). The role of homologous recombination deficiency testing in ovarian cancer and its clinical implications: do we need it? ESMO Open 6 (3), 100144. 10.1016/j.esmoop.2021.100144 34015643 PMC8141874

[B22] Otaegi-Ugartemendia M. Matheu A. Carrasco-Garcia E. (2022). Impact of cancer stem cells on therapy resistance in gastric cancer. Cancers (Basel) 14 (6), 1457. 10.3390/cancers14061457 35326607 PMC8946717

[B23] Setton J. Reis-Filho J. S. Powell S. N. (2021). Homologous recombination deficiency: how genomic signatures are generated. Curr. Opin. Genet. Dev. 66, 93–100. 10.1016/j.gde.2021.01.002 33477018 PMC8377996

[B24] Shen W. Song Z. Zhong X. Huang M. Shen D. Gao P. (2022). Sangerbox: a comprehensive, interaction-friendly clinical bioinformatics analysis platform. iMeta 1 (3), e36. 10.1002/imt2.36 PMC10989974 38868713

[B25] Smyth E. C. Nilsson M. Grabsch H. I. van Grieken N. C. Lordick F. (2020). Gastric cancer. Lancet 396 (10251), 635–648. 10.1016/S0140-6736(20)31288-5 32861308

[B26] Stewart M. D. Merino Vega D. Arend R. C. Baden J. F. Barbash O. Beaubier N. (2022). Homologous recombination deficiency: Concepts, definitions, and assays. Oncologist 27 (3), 167–174. 10.1093/oncolo/oyab053 35274707 PMC8914493

[B27] Sung H. Ferlay J. Siegel R. L. Laversanne M. Soerjomataram I. Jemal A. (2021). Global cancer statistics 2020: GLOBOCAN estimates of incidence and mortality worldwide for 36 cancers in 185 countries. Ca. Cancer J. Clin. 71 (3), 209–249. 10.3322/caac.21660 33538338

[B28] Tamai K. Nakamura-Shima M. Shibuya-Takahashi R. Kanno S. I. Yasui A. Mochizuki M. (2020). BEX2 suppresses mitochondrial activity and is required for dormant cancer stem cell maintenance in intrahepatic cholangiocarcinoma. Sci. Rep. 10 (1), 21592. 10.1038/s41598-020-78539-0 33299012 PMC7725823

[B29] Tan Y. Hu Y. Xiao Q. Tang Y. Chen H. He J. (2020). Silencing of brain-expressed X-linked 2 (BEX2) promotes colorectal cancer metastasis through the Hedgehog signaling pathway. Int. J. Biol. Sci. 16 (2), 228–238. 10.7150/ijbs.38431 31929751 PMC6949152

[B30] Telli M. L. Timms K. M. Reid J. Hennessy B. Mills G. B. Jensen K. C. (2016). Homologous recombination deficiency (HRD) score predicts response to platinum-containing neoadjuvant chemotherapy in patients with triple-negative breast cancer. Clin. Cancer Res. 22 (15), 3764–3773. 10.1158/1078-0432.CCR-15-2477 26957554 PMC6773427

[B31] Wagener-Ryczek S. Merkelbach-Bruse S. Siemanowski J. (2021). Biomarkers for homologous recombination deficiency in cancer. J. Pers. Med. 11 (7), 612. 10.3390/jpm11070612 34203281 PMC8304859

[B32] Wagner W. Kania K. D. Ciszewski W. M. (2017). Stimulation of lactate receptor (HCAR1) affects cellular DNA repair capacity. DNA Repair (Amst) 52, 49–58. 10.1016/j.dnarep.2017.02.007 28258841

