# Supplementary figures and images for: Potential value of the homologous recombination deficiency signature we developed in the prognosis and drug sensitivity of gastric cancer
Source: Front Genet. 2022 Nov 16;13:1026871. doi: 10.3389/fgene.2022.1026871 (PMC9709314; doi:10.3389/fgene.2022.1026871)

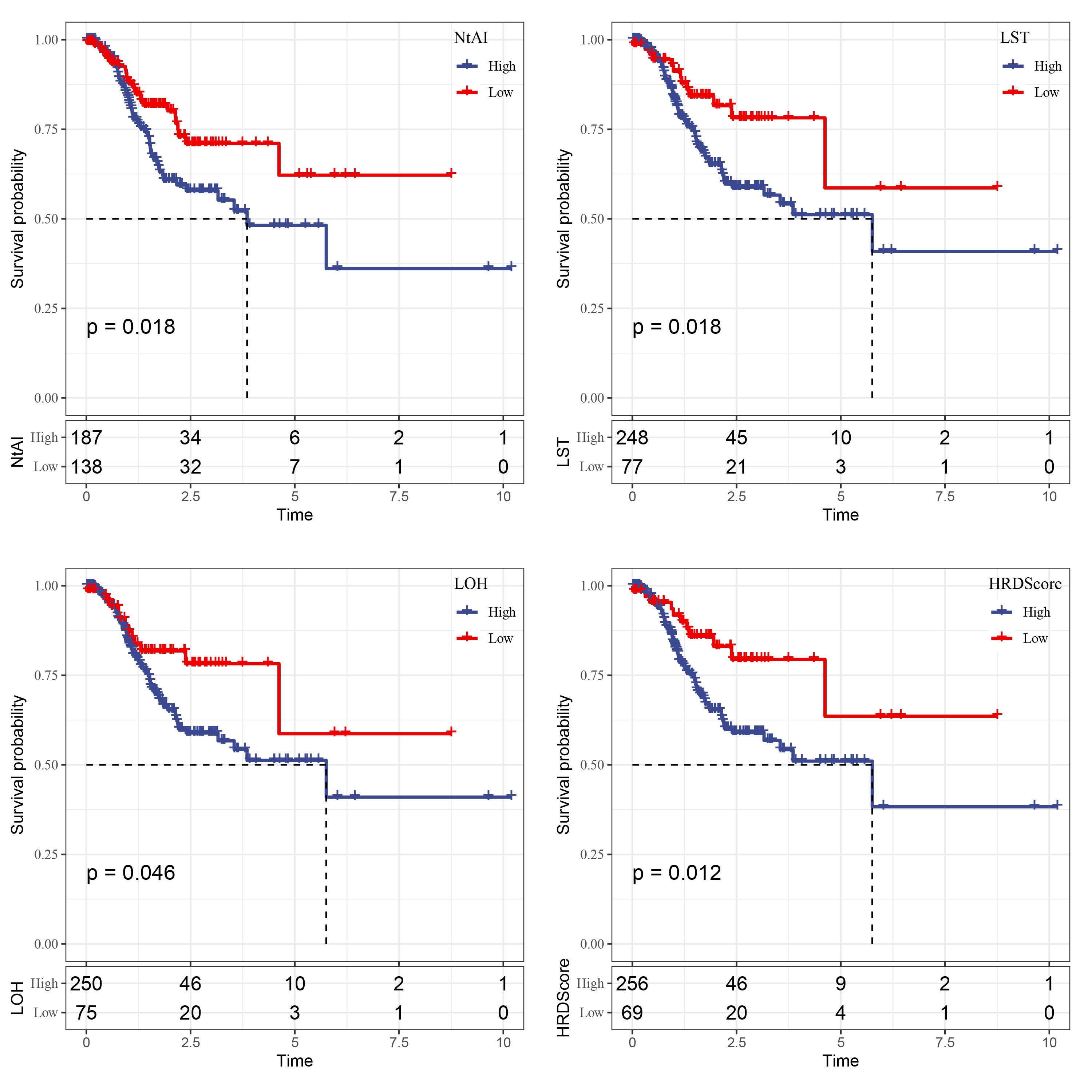

Supplement: Supplementary file 1 [file Image3.JPEG]

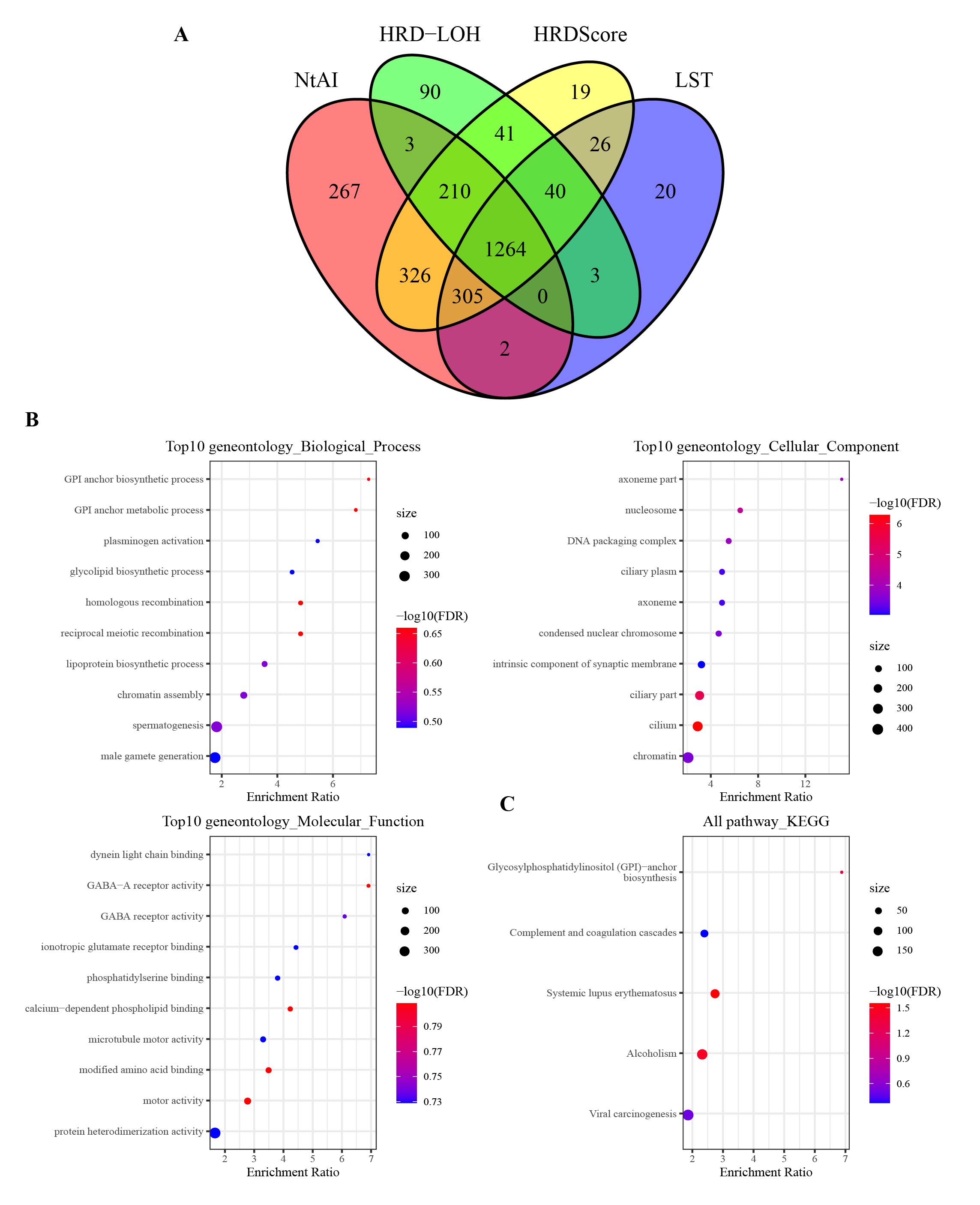

Supplement: Supplementary file 3 [file Image1.JPEG]

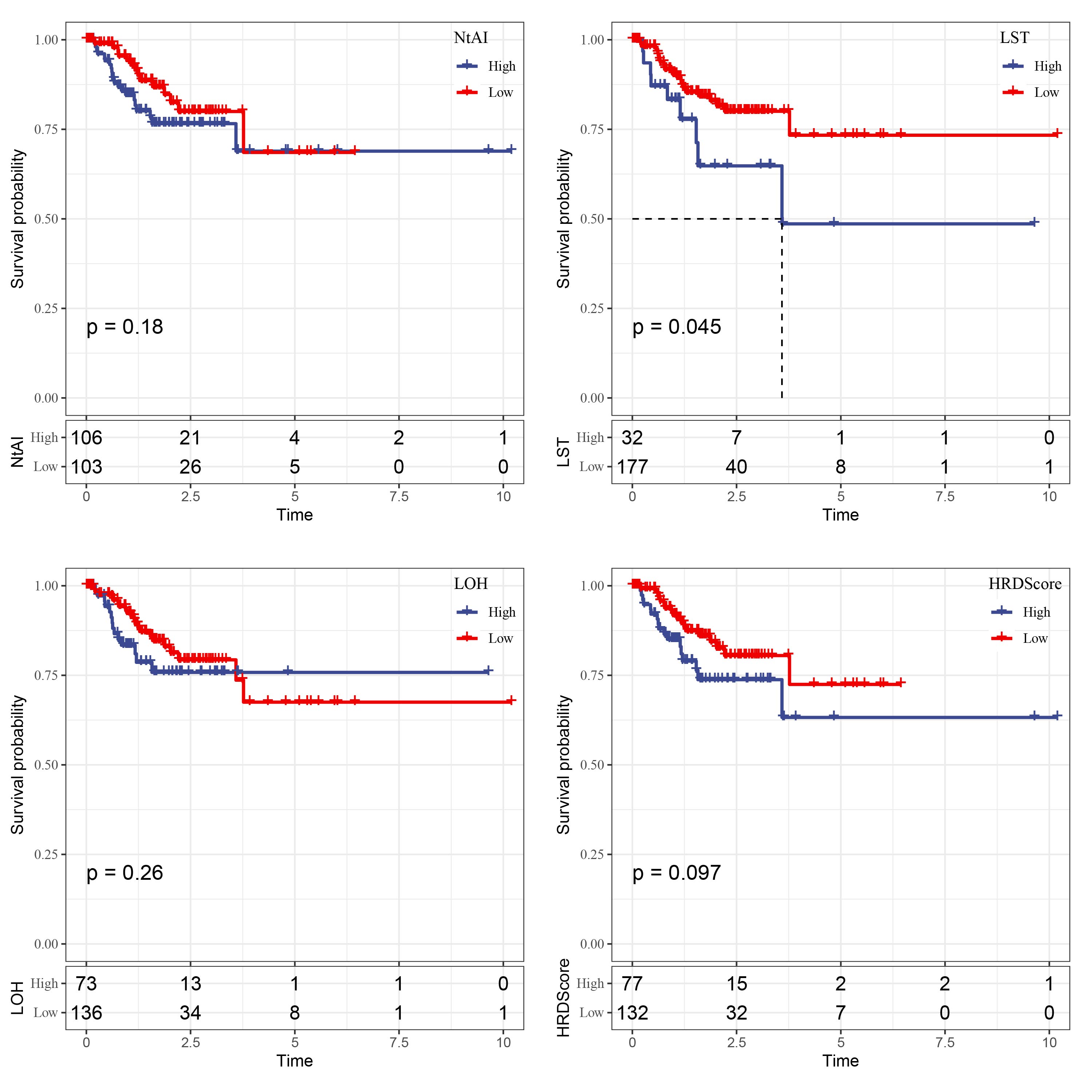

Supplement: Supplementary file 4 [file Image4.JPEG]

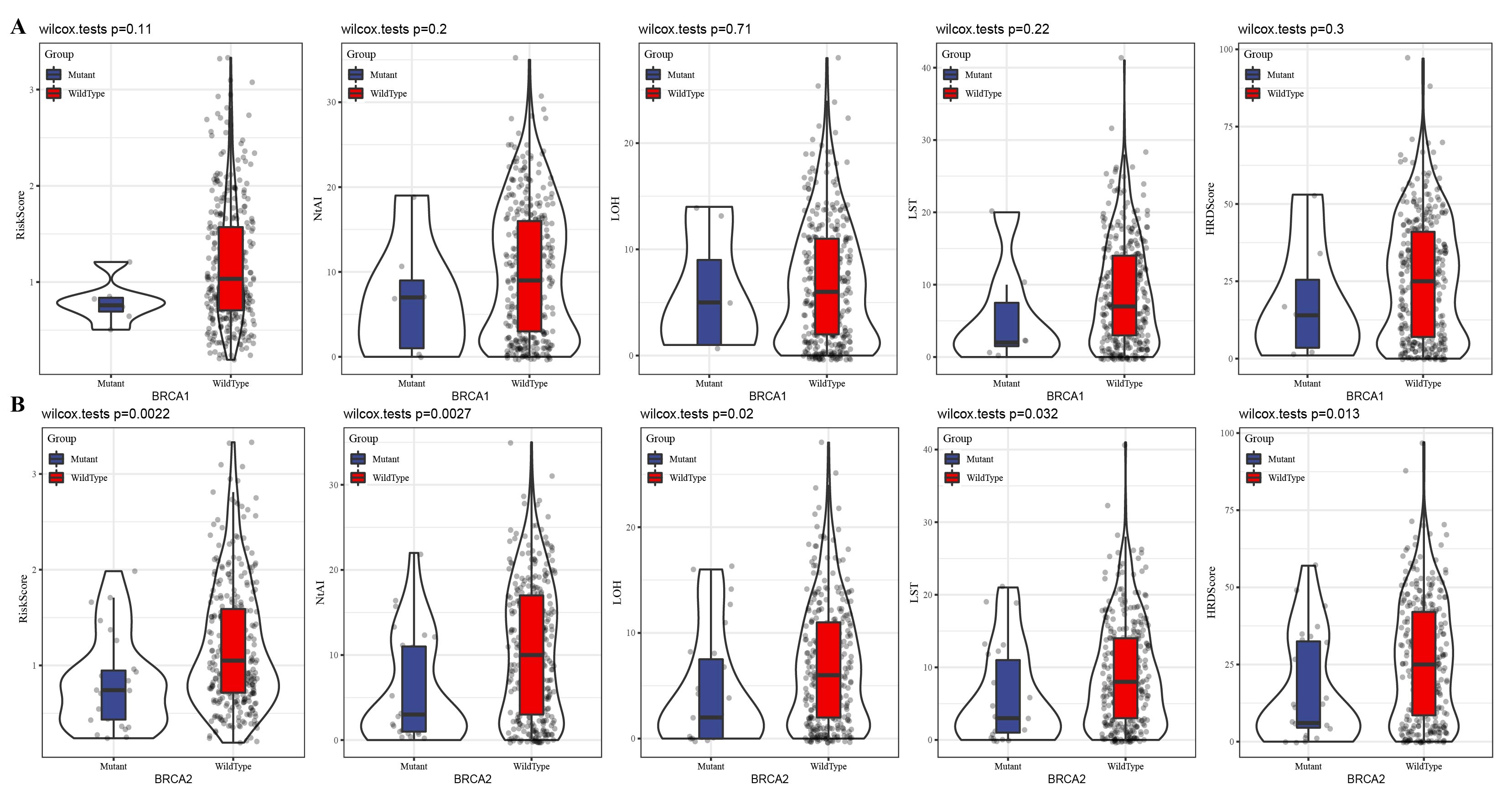

Supplement: Supplementary file 5 [file Image7.JPEG]

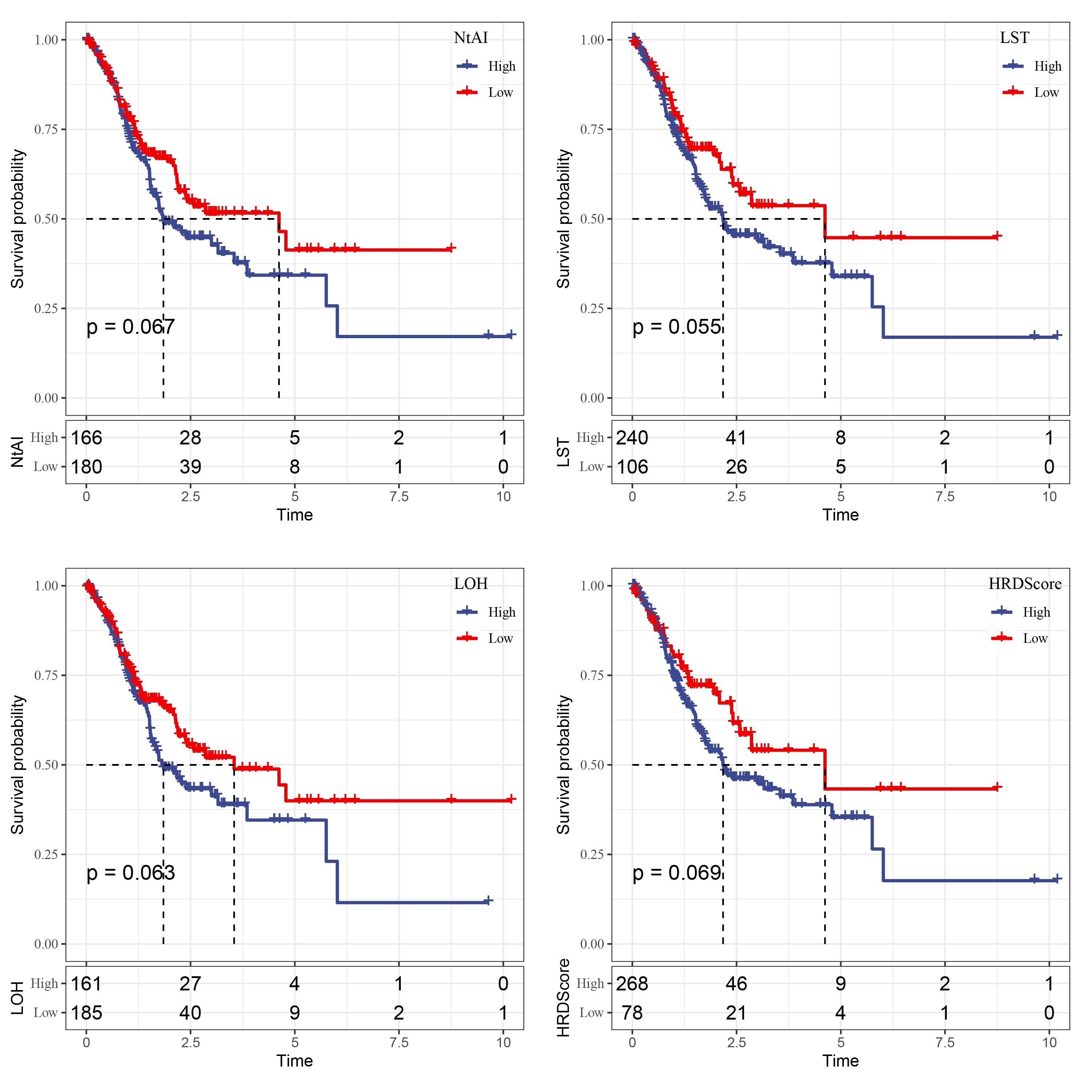

Supplement: Supplementary file 6 [file Image2.JPEG]

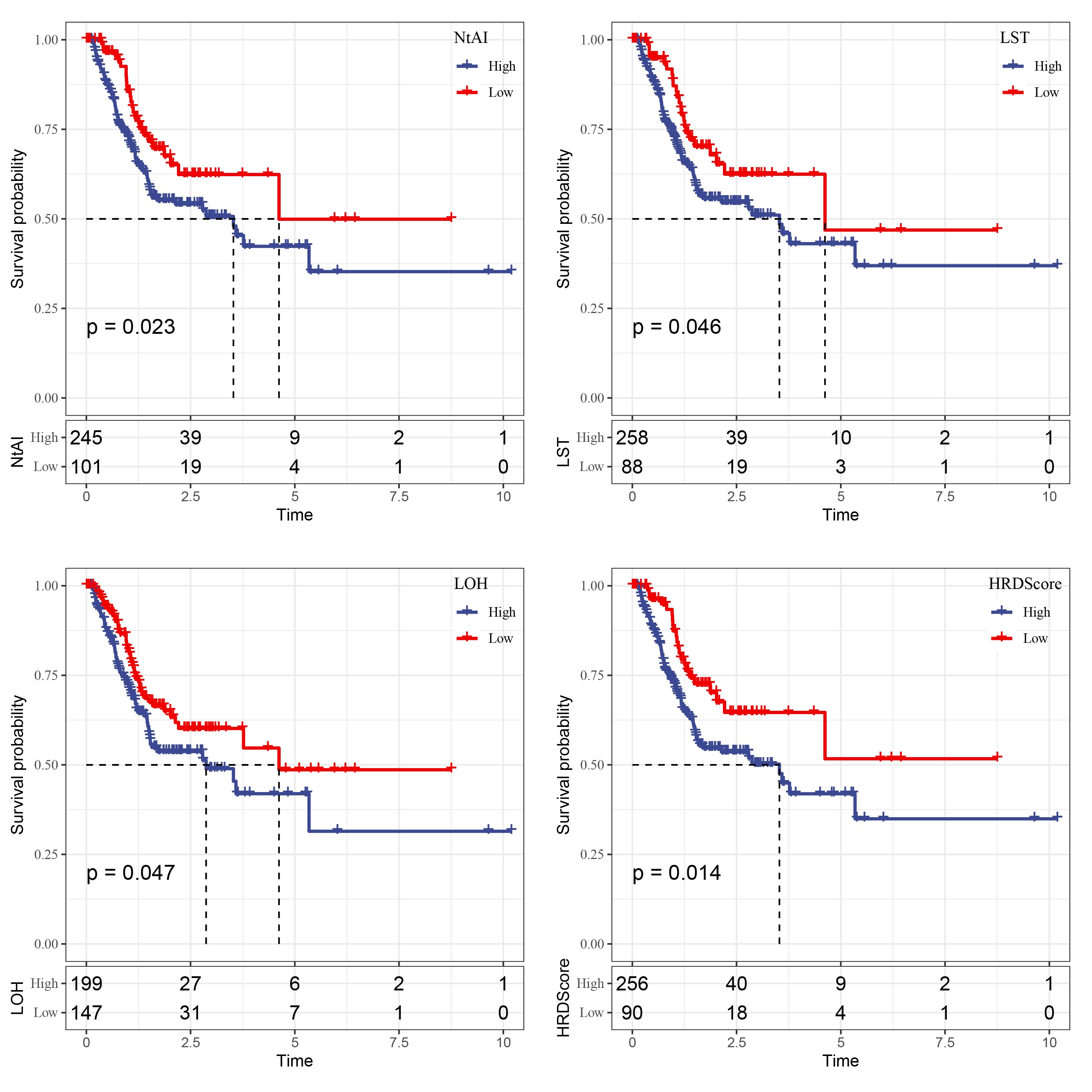

Supplement: Supplementary file 7 [file Image5.JPEG]

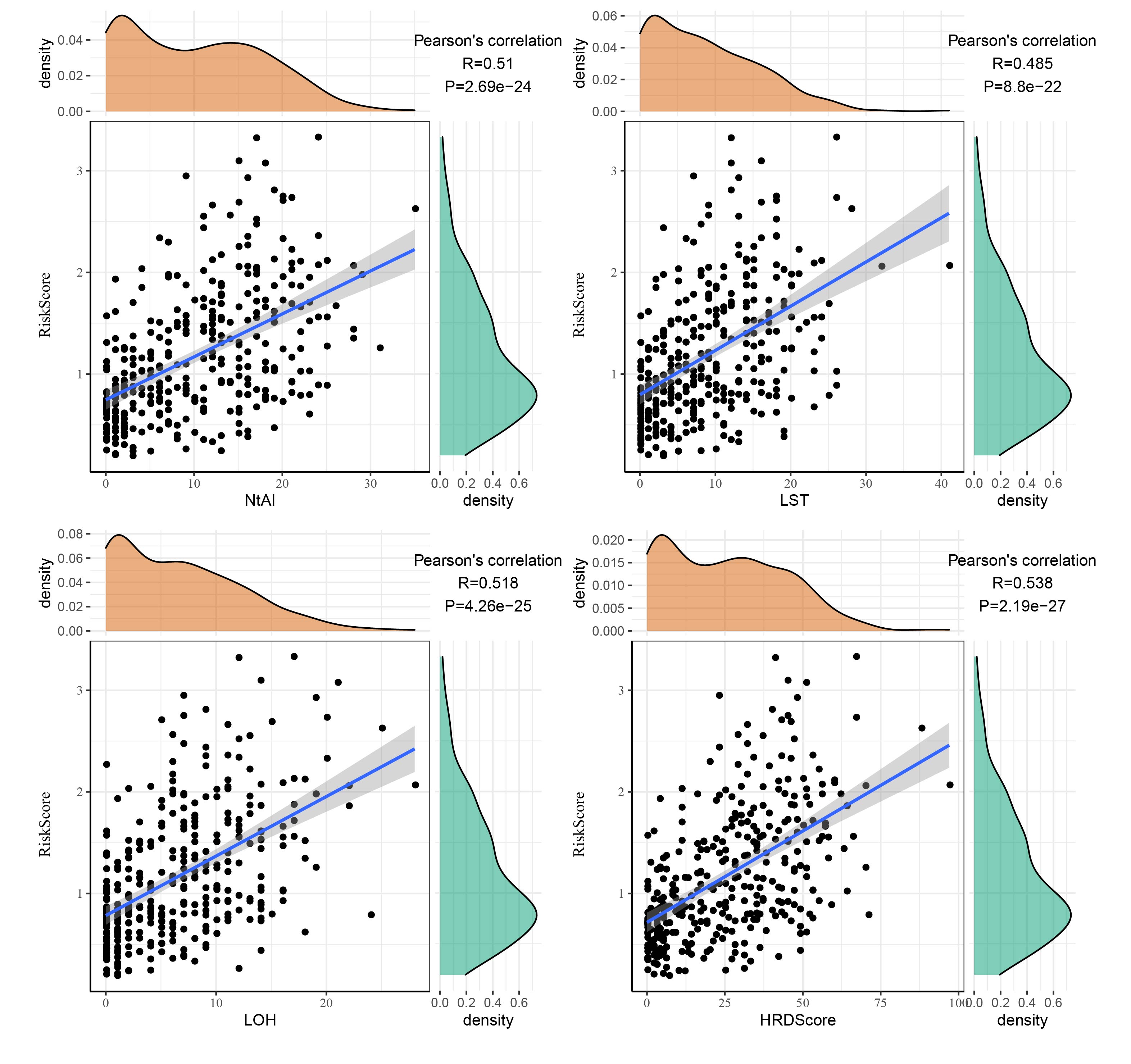

Supplement: Supplementary file 9 [file Image6.JPEG]
