# Supplementary material for: Potential value of the homologous recombination deficiency signature we developed in the prognosis and drug sensitivity of gastric cancer
Source: Front Genet. 2022 Nov 16;13:1026871. doi: 10.3389/fgene.2022.1026871 (PMC9709314; doi:10.3389/fgene.2022.1026871)
Supplement: Supplementary file 2 [file Table1.DOCX]

Data analyzed in this study:

https://www.jianguoyun.com/p/DQyJMZ0Q06bpChir0NIEIAA
