# Supplementary material for: Potential value of the homologous recombination deficiency signature we developed in the prognosis and drug sensitivity of gastric cancer
Source: Front Genet. 2022 Nov 16;13:1026871. doi: 10.3389/fgene.2022.1026871 (PMC9709314; doi:10.3389/fgene.2022.1026871)
Supplement: Supplementary file 8 [file Table3.DOCX]

**Table S1** **The sample sizes included in each cancer of TCGA.**

| Cancer type |  | Sample numbers |
| --- | --- | --- |
| ACC |  | 77 |
| BLCA |  | 396 |
| BRCA |  | 1028 |
| CESC |  | 278 |
| CHOL |  | 36 |
| COAD |  | 424 |
| DLBC |  | 46 |
| ESCA |  | 140 |
| GBM |  | 143 |
| HNSC |  | 487 |
| KICH |  | 64 |
| KIRC |  | 492 |
| KIRP |  | 278 |
| LGG |  | 499 |
| LIHC |  | 353 |
| LUAD |  | 488 |
| LUSC |  | 487 |
| MESO |  | 77 |
| OV |  | 361 |
| PAAD |  | 156 |
| PCPG |  | 160 |
| PRAD |  | 470 |
| READ |  | 155 |
| SARC |  | 242 |
| SKCM |  | 102 |
| STAD |  | 346 |
| TGCT |  | 133 |
| THCA |  | 459 |
| THYM |  | 102 |
| UCEC |  | 525 |
| UCS |  | 54 |
| UVM |  | 79 |
